# Supplementary material for: A Systematic Review of Sexual Minority Women’s Experiences of Health Care in the UK
Source: Int J Environ Res Public Health. 2019 Aug 21;16(17):3032. doi: 10.3390/ijerph16173032 (PMC6747244; doi:10.3390/ijerph16173032)
Supplement: Supplementary file 1 [file ijerph-16-03032-s001.pdf]

Supplement to Meads et al, A systematic review of sexual minority women's experiences of health care in the UK

References to included studies

1. Almack, K.; Seymour, J.; Bellamy, G. Exploring the impact of sexual orientation on experiences and concerns about end of life care and on bereavement for lesbian, gay and bisexual older people. *Sociology* **2010**, *44*, 908–924.
2. Balding, A. Young people in Cambridgeshire schools, the health-related behaviour survey 2014, a report for LGBT. The Schools Health Education Unit: Exeter, UK, 2014.
3. Bristowe, K.; Hodson, M.; Wee, B.; Almack, K.; Johnson, K.; Daveson, B.A.; Koffman, J.; McEnhill, L.; Harding, R. Recommendations to reduce inequalities for LGBT people facing advanced illness: ACCESSCare national qualitative interview study. *Palliat. Med.* **2018**, *32*, 23–35.
4. Carter, L.; Hedges, L.; Congdon S. Using diversity interventions to increase cervical screening of lesbian and bisexual women. *J. Psychol. Issues Organ. Cult.* **2013**, *3*, 133–145.
5. Cherguit, J.; Burns. J.; Pettle, S.; Tasker, F. Lesbian co-mothers' experiences of maternity healthcare services. *J. Adv. Nurs.* **2013**, *69*, 1269–1278.
6. Elliott, M.N.; Kanouse, D.E.; Burkhart, Q.; Abel, G.A.; Lyratzopoulos, G.; Beckett, M.K.; Schuster, M.A.; Roland, M. Sexual minorities in England have poorer health and worse health care experiences: A national survey. *J. Gen. Intern. Med.* **2014**, *30*, 9–16.
7. Evans, M.; Barker, M. How do you see me? Coming out in counselling. *Br. J. Guid. Counc.* **2010**, *38*, 375–391.
8. Fenge, L-A. Developing understanding of same-sex partner bereavement for older lesbian and gay people: Implications for social work practice. *J. Gerontol. Soc. Work 2014, 57:2–4*, 288–304.
9. Fish, J. Coming out about breast cancer: Lesbian and bisexual women. Policy and practice implications for cancer services and social care organisations. De Montfort University: Leicester, UK, 2010.
10. Fish, J; Bewley, S. Using human rights-based approaches to conceptualise lesbian and bisexual women's health inequalities. *Health Soc. Care Community* **2010**, *18*, 355–362.
11. Fish, J.; Williamson, I. Exploring lesbian, gay and bisexual patients' accounts of their experiences of cancer care in the UK. *Eur. J. Cancer Care* 2016, doi:10.1111/ecc.12501.
12. Formby, E. Sex and relationships education, sexual health, and lesbian, gay and bisexual sexual cultures: Views from young people. *Sex. Educ.: Sex., Soc. Learn.* **2011**, *11*, 255–266.
13. Formby, E. Lesbian and bisexual women's human rights, sexual rights and sexual citizenship: Negotiating sexual health in England. *Cult. Health Sex.* **2011**, *13*, 1165–1179.
14. Government Equalities Office (GEO). National LGBT survey research report. UK Government Department for Education: Manchester, UK, 2018.
15. Guasp, A. Lesbian gay and bisexual people in later life. Stonewall: London, UK, 2011.
16. Humphreys, S.; Worthington, V. Best practice in providing healthcare to lesbian, bisexual and other women who have sex with women. National LGBT Partnership, UK, 2016.
17. Ingham, C.F.; Eccles, F.J.; Armitage, J.R.; Murray, C.D. Same-sex partner bereavement in older women: An interpretative phenomenological analysis. *Aging Ment. Health 2016*, doi:10.1080/13607863.2016.1181712.
18. Knocker, S. Perspectives on ageing: Lesbians, gay men and bisexuals. Joseph Rowntree Foundation: West Sussex, UK, 2012.
19. Lee, E.; Taylor, J.; Raitt, F. 'It's not me, it's them': How lesbian women make sense of negative experiences of maternity care: A hermeneutic study. *J. Adv. Nurs.* **2010**, *67*, 982–990.
20. Light, B.; Ormandy, P.; Bottomley, R.; Emery, A. Lesbian, gay & bisexual women in the north west: A multi-method study of cervical screening attitudes, experiences and uptake. University of Salford, LGF: Manchester, UK, 2011.
21. Macredie, S. The challenge for change. Health needs of lesbian, gay and bisexual people in Bradford and district. Equity Partnership: Bradford, UK, 2010.
22. McDermott, E.; Hughes, E.; awlins, V. Queer futures final report. Understanding lesbian, gay, bisexual and trans (LGBT) adolescents' suicide, self-harm and help-seeking behaviour. Department of Health Policy Research Programme: London, UK, 2106.

23. Price, E. Coming out to care: Gay and lesbian carers' experiences of dementia services. *Health Soc. Care Community 2010, 18:*160–168.
24. Price, E. Gay and lesbian carers: Ageing in the shadow of dementia. *Ageing Soc.* **2012**, *32*, 516–532.
25. River, L. Appropriate Treatment Older lesbian, gay and bisexual people's experience of general practice. Age of Diversity and Polari: London, UK, 2011.
26. Urwin, S.; Whittaker, W. Inequalities in family practitioner use by sexual orientation: Evidence from the English General Practice Patient Survey. *BMJ Open 2016*, 6, e011633.
27. Westwood, S. 'We see it as being heterosexualised, being put into a care home': Gender, sexuality and housing/care preferences among older LGB individuals in the UK. *Health Soc. Care Community 2016, 24,* e155–e163.
28. Westwood, S. Dementia, women and sexuality: How the intersection of ageing, gender and sexuality magnify dementia concerns among lesbian and bisexual women. *Dementia* **2016**, *15*, 1494–1514.
29. Willis, P.; Ward, N.; Fish, J. Searching for LGBT carers: Mapping a research agenda in social work and social care. *Br. J. Soc. Work* **2011**, *41*, 1304–1320.
